# Therapeutic perspectives for prion diseases in humans and animals

**DOI:** 10.1371/journal.ppat.1012676

**Published:** 2024-12-10

**Authors:** Rebeca Benavente, Rodrigo Morales

**Affiliations:** 1 Department of Neurology, McGovern Medical School, The University of Texas Health Science Center at Houston, Houston, Texas, United States of America; 2 Centro Integrativo de Biologia y Quimica Aplicada (CIBQA), Universidad Bernardo O’Higgins, Santiago, Chile; National Institutes of Health, UNITED STATES OF AMERICA

## Introduction

Prion diseases are a group of fatal neurodegenerative disorders affecting several mammals including humans, cattle, cervids, sheep, goats, and others [1–3]. These diseases are driven by the misfolding of the cellular prion protein (PrP^C^) into β-sheet rich structures. The disease-associated prion, termed PrP^Sc^, is prone to aggregation as well as neurotoxic [4–6]. Prion diseases can be associated with mutations in the prion protein gene (*PRNP*), induced by the exposure to prion-contaminated materials, or arise sporadically [3,7].

A relevant area of research in the prion field is drug discovery. Despite many efforts spanning decades, prion diseases still have no significant modifying treatments or cures [1,2,7–10]. Several factors affect the development of effective anti-prion treatments including the different conformations that PrP^Sc^ particles acquire (also known as prion strains [7,11]), the limited number of prion strains that are available to be studied in cell-culture models [1], the rare incidence of human prion diseases that limit patient recruitment, and the still elusive understanding of prion pathogenesis, among others [7,12]. Moreover, the lack of sensitive detection methods to identify the disease in its early stages [13] makes it hard to treat individuals before the onset of symptoms when brain damage is already extensive and likely irreversible [3].

This article focuses on the different approaches used to develop anti-prion therapies targeting PrP^C^, PrP^Sc^, and associated pathological pathways. We also discuss methods that have been employed in the field to develop and evaluate new therapies. Finally, we discuss some therapeutic strategies tested in humans and animals, as well as future perspectives for therapies against prion diseases.

## PrP^Sc^-specific anti-prion strategies

**Fig 1:**
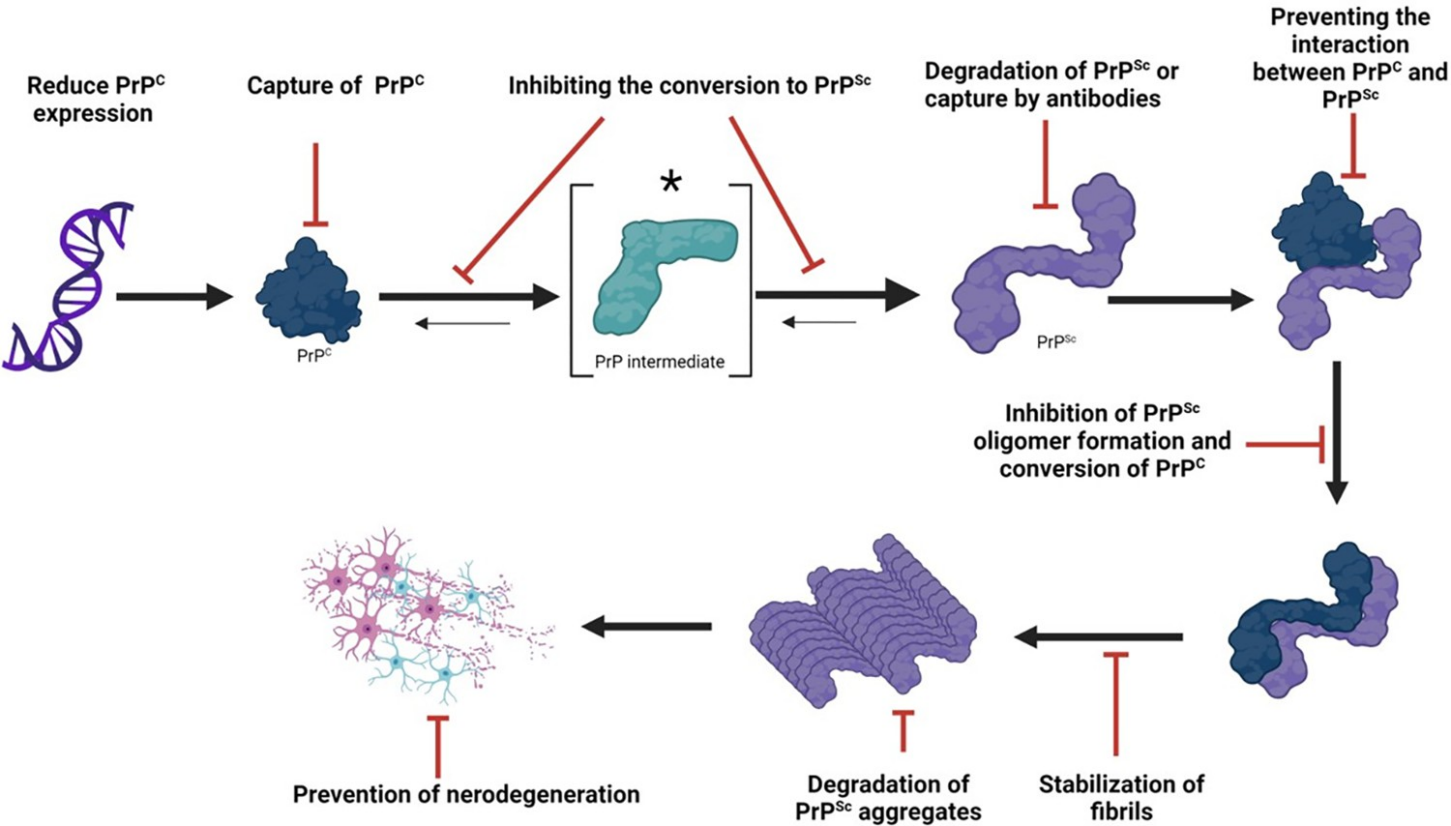
PrP-specific therapeutic strategies against prion diseases. Different strategies have been explored targeting either PrP^C^ or PrP^Sc^. Some approaches have focused in knocking down or capturing PrP^C^ to limit the availability of substrate for the prion protein misfolding process. Other strategies have been used to directly inhibit the PrP^C^-to-PrP^Sc^ conversion process. Additional avenues, either capturing or degrading PrP^Sc^, have also been tested. Along this line, further approaches aim to inhibit the formation of the most deleterious prion particles (e.g., oligomers). Moreover, increasing the degradation of PrP^Sc^ aggregates has also been tested. This figure was created using BioRender.

The key elements for the progression of prion disease in a given host include PrP^C^ and PrP^Sc^. Most therapeutic strategies have attempted to target either PrP^C^, PrP^Sc^, or both at the different steps of the protein misfolding process [2,4–10,12,13]. Consequently, therapeutic strategies explored against prion diseases include the reduction of the *PRNP* expression, stabilization or capture of PrP^C^, use of antibodies against either normally folded or misfolded forms of the prion protein, blocking the conversion of PrP^C^ into PrP^Sc^, inhibition of PrP^Sc^ aggregation or disaggregation of existing fibrils, among others [2,3,7,8] (Fig 1). Other, more unconventional strategies, such as vaccinations and prion-interference approaches have also been attempted [8,10,13–16]; however, the existence of different conformations of prions (prion strains) suggests that these strategies may be limited to a narrow (or even a single) range of prion agents [1,7,8,17]. From the abovementioned strategies, the most promising results have been obtained by reducing or eliminating the expression of PrP^C^ [18]. It has been extensively demonstrated that halting the expression of PrP^C^ provides resistance to prion infection in multiple systems including cell-free assays, cell cultures, rodent models, and large animals [3,7,8,18]. Along this line, treatment with interference RNA, antisense oligonucleotides, and antibodies targeting the prion protein have provided promising results as evaluated by the substantial increase in the survival times of experimental subjects or preventing the disease from occurring [2,4,7,10,11]. Importantly, the lack of PrP^C^ expression appears to be safe, although additional studies need to be performed in this direction [3,7,11]. Along this line, a recently described epigenetic editor able to silence the expression of PrP^C^ holds promise as a potential therapy against prion diseases [19].

Complementary, many small molecules targeting PrP^Sc^ have been tested in experimentally or naturally afflicted subjects with variable results, mostly unsuccessfully [7,8,10,12]. Nevertheless, strategies targeting PrP^Sc^ are predicted to be of capital importance in clinical cases where the accumulation of infectious particles in the brain is already established [18]. Published evidence demonstrates multiple challenges arising from these research efforts [12]. These include the prion strain-specific targeting of anti-prion molecules, and the selection of drug-resistant prion strains [1,3,11]. Fortunately, the development of in vitro prion amplification methods holds promise for the screening of large compound libraries that may assist in the identification of strain-specific anti-prion molecules [1,8,12,17]. Nevertheless, it is important to consider their intrinsic limitations (e.g., no evaluation of biological functions). The advantages and disadvantages of methods used in the research of anti-prion therapies are summarized in Table 1.

**Table 1:** Conventional and emerging methods employed for drug discovery in prion diseases.

Approach	Advantages	Disadvantages
**Bioassays**	• Most reliable method to study prion therapeutics • Allows to analyze the whole spectrum of mechanisms associated with prion therapeutics • Provide data on pharmacokinetics and potential side effects • Maintain prion strain properties • The availability of transgenic mice expressing the prion protein of different animal species allows studies applied for relevant prion strains.	• Long incubation periods • Expensive • Bioethical concerns • Not suitable for large screenings
**Cell cultures**	• Relatively simple • Allow the study of intracellular mechanisms • Allow for toxicity assessment	• Available for a limited number of prion strains
**Protein misfolding cyclic amplification (PMCA)**	• Cost effective • Ultra-sensitive • Maintain strain properties • Potential for high-throughput analyses	• Only allows the study of protein–compound interactions
**Real-time quacking induced conversion (RT-QuIC)**	• Cost effective • Ultra-sensitive • Potential for high-throughput analyses	• Products do not maintain relevant biological properties • Fluorophore interactions may interfere with the reading of certain molecules • Only allows the study protein–compound interactions • Do not maintain strain properties
**In silico**	• Suitable for high-throughput analyses • Allows for understanding the mechanism of action of the studied molecules	• Limited by the difficulties posed to solve the structure of PrP^Sc^ strains • Does not allow to assess biological interactions
**Human brain organoids**	• Predict the neurotoxicity of drugs • Allows to analyze a wider spectrum of mechanisms associated with prion therapeutics • Can be coupled with other in vitro techniques	• Not suitable for high-throughput screening • Since there is no vasculature, the penetration of drugs might be reduced • Do not fully recapitulate the brain structure (brain regions) • Potential variability between organoids

## Strategies targeting PrP^Sc^-associated toxic mechanisms

**Fig 2:**
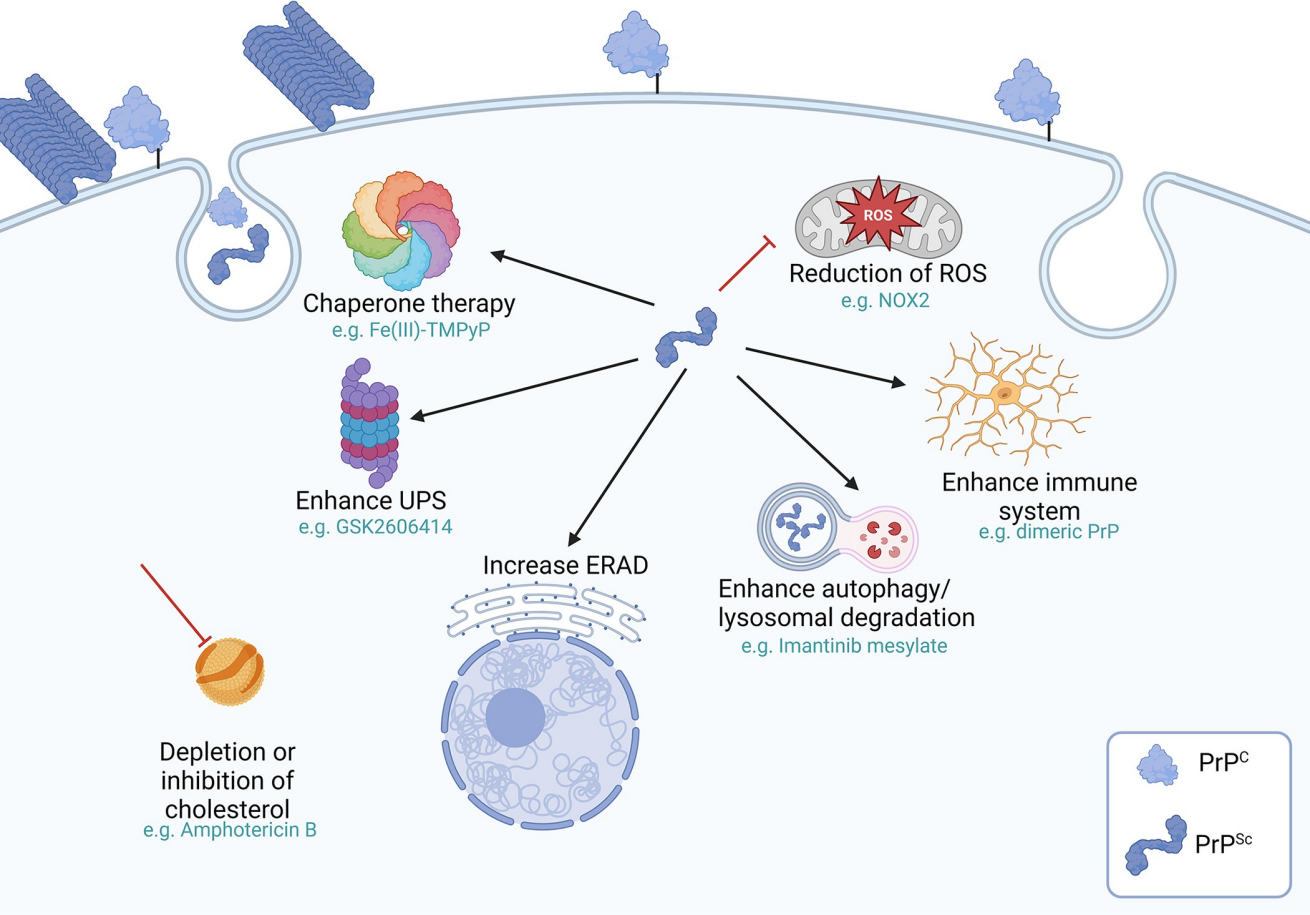
PrP downstream approaches for anti-prion therapies. The prion misfolding process can be reduced by preventing the localization of the normal prion protein in lipid rafts (one of the hypothesized sites of conversion) through the depletion of cholesterol. Prion toxicity can also be reduced by targeting its extensively described downstream mechanisms, including apoptosis mediated by endoplasmic reticulum or mitochondrial stresses. Attempts to increase the degradation of this protein through autophagy, lysosomal degradation and priming of the immune system have also been explored. Some reports also suggest that the increase of endoplasmic reticulum-associated protein degradation (ERAD) pathways might be beneficial to the degradation of PrP^Sc^. An additional therapeutic option involves the refolding of the prion protein into its native state through chaperones. Finally, the prevention of neurodegeneration by reducing ROS production has also been explored. Some of the drugs tested in different pathways are marked in this figure. This figure was created using BioRender.

Anti-prion therapies have also been designed, or have been serendipitously found, to target elements promoting the formation of, or activated by, PrP^Sc^ (Fig 2). Some of these targets correspond to metabolites acting at intracellular processes. While some of them promote the degradation and clearance of PrP^Sc^/PrP^C^ through different pathways (e.g., autophagy, ubiquitin-proteasome system) [1,2,7,10,11], other potential therapies act by preventing the damage generated by the reactive oxygen species (ROS) induced by PrP^Sc^ (by either decreasing their load or providing resilience to brain cells [2,10]). Other molecules have been shown to stimulate the immune system, by priming it to ameliorate neurodegeneration [2,15]. Although many of these approaches have shown promise in cell culture systems, they have been moderately effective or not effective at all in bioassays [10]. Moreover, some of these downstream mechanisms, such as the endoplasmic reticulum associated degradation, have been proven to be important for the clearance of both PrP^Sc^ and PrP^C^ under physiological and pathological conditions [7]. Unfortunately, it is unclear the overall impact of these therapies considering the continuous formation of PrP^Sc^ assemblies that are expected to keep contributing to the pathological and clinical progression in afflicted individuals. Additional insights on this subject can be followed in [2].

## Efforts to test therapeutic strategies in natural hosts and perspectives

Multiple drugs, including small molecules and antibodies, have been studied in naturally infected subjects, including humans and cervids. Most of these efforts resulted in either modest or no increase in survival or improvements in clinical signs. In humans, this was the case of quinacrine, amantadine, or flupirtine [1,8,18]. All these molecules, despite showing promising results in cell cultures and animal models infected with rodent prions, were inefficient in human subjects [3,8,13]. One hypothesis is that these drugs interacted with the specific conformation present in laboratory prions, but were inefficient against human prion strains. It is worth noting that only flupirtine was tested in a controlled randomized trial [7,8,13]. Other preclinical trials, such as those described for doxycycline, have shown some moderate effects mainly by extending the expected survival of patients intervened at early clinical stages of Creutzfeldt–Jakob disease [1,8]. A similar scenario has been described for the anti-PrP antibody PRN100. There, the number of patients treated was low and the treatment had to be suspended in some patients due to exhaustion of the antibody [7–9]. Considering these limitations, it is hard to assess the real impact of this passive vaccination strategy. Currently, the antisense oligonucleotide (ASO) ION717, designed for genetic cases of prion diseases, is in Phase 1/2a (https://clinicaltrials.gov/study/NCT06153966).

In animals, 3 different vaccines have been tested in chronic wasting disease infected cervids. In general, they all showed limited therapeutic effects. One of them was based on a recombinant version of the prion protein. Unfortunately, trials using this technology protected a single subject [14]. Another immunization effort included an attenuated salmonella strain expressing PrP. This trial was experimentally conducted in CWD infected cervids and demonstrated increased incubation periods in the immunized subjects after 8 doses [20]. Nonetheless, these vaccinations required multiple doses which could be difficult to achieve, especially in wild settings. A third vaccine strategy, specifically designed to target PrP^Sc^, was conducted in experimentally infected elk and showed an accelerated onset of the disease. The latter suggested that PrP^Sc^ targeting can lead to worse outcomes [21]. However, the molecular mechanisms leading to these unexpected results need to be further evaluated.

Perhaps, the most promising therapeutic approach for prion diseases involves the reduction or knockdown of PrP^C^ by antisense oligonucleotides. This has been shown as an effective strategy in animal models as it can increase survival by 98% [4]. This is one of the most beneficial effects shown yet. In addition, knocking down PrP^C^ appears as a universal strategy in the context of different prion strains.

It is relevant to note that despite promising results, all these therapies do not appear as definitive cures for these devastating diseases. For example, the effectiveness of PrP^C^ reduction in subjects with established pathology is still contentious and this avenue seems more feasible in individuals with genetic risks (*PRNP* mutations) or at early (most likely, preclinical) stages of the disease. Along this line, a PrP^C^/PrP^Sc^-focused, combinatory therapy will likely be able to provide the most efficient results in clinical cases, since the simultaneous targeting of PrP^C^ and PrP^Sc^ should prevent the formation of new prion propagons while clearing the existing ones. However, we must also consider the potential adaptation or selection of drug-resistant prion strains in these processes [1,3,11,22]. Hence, this should be accompanied by continuous monitoring to identify the predominant prion strains present across the process and adapt the treatment, accordingly. It is also clear that complementation with therapies targeting downstream events (e.g., cellular toxicity) will also provide protection against ongoing damage in clinical patients. Future research is expected to clarify these matters and hopefully identify a cure for these still fatal group of diseases.
